# Weighing the risk of GLP-1 treatment in older adults: Should we be concerned about sarcopenic obesity?

**DOI:** 10.1016/j.jnha.2025.100652

**Published:** 2025-08-16

**Authors:** Konstantinos Prokopidis, Robin M. Daly, Charlotte Suetta

**Affiliations:** a Department of Musculoskeletal Ageing and Science, Institute of Life Course and Medical Sciences, University of Liverpool, Liverpool, United Kingdom; b Institute for Physical Activity and Nutrition, School of Exercise and Nutrition Sciences, Deakin University, 221 Burwood Highway, Burwood, Melbourne, Victoria, Australia; c Department of Clinical Medicine, Faculty of Health, University of Copenhagen, Copenhagen, Denmark; d Department of Geriatric and Palliative Medicine, Copenhagen University Hospital – Bispebjerg and Frederiksberg, Copenhagen, Denmark

**Keywords:** Weight loss, Sarcopenic obesity, GLP-1 receptor agonists, Weight cycling, Ageing

## Abstract

Glucagon-like peptide-1 receptor agonists (GLP-1 RAs) have been pivotal for obesity treatment, achieving 15–25 % weight loss over 12–24 months, but questions remain about the potential influence of concomitant losses in muscle mass. Furthermore, low adherence, driven by high costs and side effects, results in up to two-thirds of users discontinuing treatment within a year, although up to a half reinitiate treatment. Given that cessation of treatment often leads to significant weight regain, there are concerns that older adults may be at risk for sarcopenic obesity; a condition characterized by excessive adiposity and low skeletal muscle mass which is prevalent in 10–20 % of older adults. The risk for sarcopenic obesity may be further exacerbated by weight cycling related to repeated treatment cessation which may concomitantly exacerbate fat mass gains while reducing muscle mass. This mini-review examines the risk of sarcopenic obesity as an unintended consequence of GLP-1 RA cessation in older adults, highlighting the need for raising awareness and preventative strategies.

## 1 Introduction

Glucagon like peptide-1 receptor agonist (GLP-1 RAs) therapies can elicit clinically meaningful weight loss (∼15–25%) [1] that has been associated multiple cardiometabolic benefits. As a result, prescription of GLP-1 RAs has increased dramatically, and in the US alone, ∼2 million people were prescribed GLP-1 based medications from 2018 to 2024 [2]. However, a key challenge is that adherence is often low, which has been related to high costs and side-effects. For instance, a study of 125,475 adults with or without type 2 diabetes revealed that 46–65 % receiving GLP-1 RAs discontinued their prescription within 12-months, but 36–47 % reinitiated treatment [3]. These findings could raise several concerns. First, cessation of GLP-1 treatment is associated with marked weight regain, as highlighted in a recent meta-analysis of eight randomised controlled trials which reported that participants taking semaglutide/tirzepatide regained 9.69 kg (95% CI 5.78, 13.60) over 48–52 weeks [4]. Second, weight cycling (weight loss followed by weight regain) may result in gains in fat mass that are accompanied by losses (and no subsequent regain) in muscle mass [5]. Given that use of GLP-1 RAs have been associated with muscle loss, an important question that remains is whether repeated GLP-1 RA use and cessation alter body composition in favour of fat mass relative to lean mass, pushing individuals with obesity closer to sarcopenic obesity. This mini-review aims to examine the potential emergence of sarcopenic obesity as an unintended consequence of GLP-1 RA cessation.

## 2 Sarcopenic obesity

Sarcopenic obesity is a condition characterized by the simultaneous presence of excessive adiposity and low muscle mass and/or function [6] that is estimated to affect 10–20 % of older adults, depending on the diagnostic criteria [7]. This dual phenotype has detrimental effects on both clinical and patient-centred outcomes, including functional limitations and increased morbidity risk (e.g., type 2 diabetes, fractures), contributing to lower quality of life and survival rates [8]. Consequently, it is important to raise awareness of this condition, identify those at risk and ensure comprehensive preventative strategies are implemented early to mitigate its onset and progression, particularly in at-risk populations such as older adults with obesity and low muscle mass.

## 3 Weight loss and weight cycling as risk factors for sarcopenic obesity

Weight loss interventions can improve body composition and cardiometabolic profiles in adults with obesity, but an ongoing concern is the concomitant loss in muscle mass. For instance, a previous 12-month calorie restriction (CR) trial in adults 50–60 years that resulted in a mean ± SE ∼10.7 ± 1.4% reduction in weight demonstrated significant losses in thigh muscle volume (−6.9 ± 0.8%) and knee flexion strength (−7.3 ± 3.0%) [9]. Similarly, in middle-aged obese adults a mean ± SD ∼21.7 ± 13.9% loss in fat-free mass (FFM) as part of total weight loss was found at 2-years post-bariatric surgery [10]. These muscle losses can occur rapidly and are associated with the magnitude of weight loss. In a study of 3596 patients (20% males, mean age 43.5 years) receiving sleeve gastrectomy or Roux-en-Y gastric bypass surgery followed for 3-years it was found that the highest rate of loss in FFM occurred at 3- and 6-months (57% and 78% of peak loss) post-surgery, with higher preoperative BMI one of the key factors associated with greater FFM loss [11]. Of clinical relevance is that about 30–35% of weight lost is typically regained within 12-months of surgery, and by the fifth year, half of the patients revert to their original weight [12]. Collectively, these findings highlight that many overweight/obese adults experience weight cycling, where repeated intentional weight loss and unintentional regain could adversely disrupt body composition.

Weight cycling has been linked to lower lean mass and muscle strength, particularly in those who experience multiple cycles (≥6 times) [13]. A 2025 review on the effects of weight cycling on muscle and sarcopenia suggested that it may lead to disproportionate fat regain, reduced FFM, accelerated age-related muscle loss and an increased risk for functional impairment and disability, particularly in older or frail individuals, contributing to worsened muscle health outcomes including sarcopenic obesity [14]. Therefore, given the potential for increased risk of fat mass gain, persistent muscle loss, and declining physical capacity with weight cycling, coupled with evidence that the prevalence of sarcopenic obesity is estimated to be ∼20% in obese treatment-seeking adults [15], it is possible voluntary weight loss followed by involuntary regain may increase (or further exacerbate) the risk of sarcopenia and sarcopenic obesity, particularly in older adults.

## 4 GLP-1 receptor agonist cessation

**Fig. 1:**
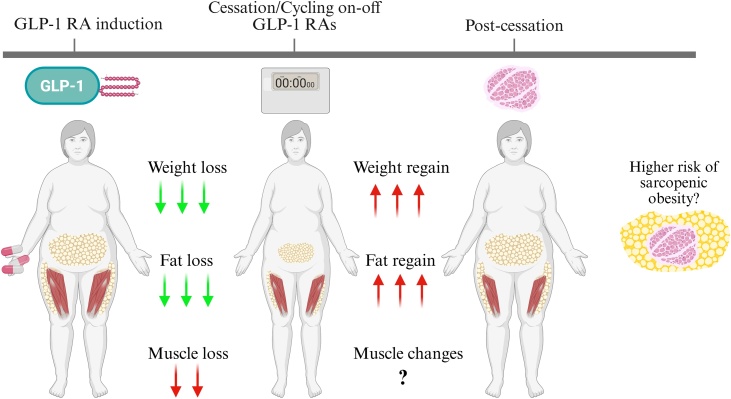
Illustration of weight regain following GLP-1 RA cessation and the potential risk for sarcopenia and sarcopenic obesity in individuals with obesity.

While cessation of GLP-1 RA treatment is associated with marked weight regain, whether repeated GLP-1 RAs treatment and subsequent weight regain increases the risk for sarcopenia or sarcopenic obesity remains unknown. Based on previous weight cycling studies, there is the potential that rapid weight regain may promote a higher proportion of adipose tissue accumulation compared to lean mass. A study examining body composition changes 5-years post-semaglutide found those who gained weight displayed no significant changes in muscle area (188.9→198.4 cm^2^) at the lumbar spine (L3)-level, but gained visceral (313→334 cm²), subcutaneous (449→486 cm²), and intermuscular fat (37.6→48.4 cm²), and experienced decreased muscle attenuation (13.1→5.9 HU), indicating higher muscle fat infiltration [16]. In this context, previous studies have shown that fat mass may be regained to a greater extent than lean mass up to 12-months following intentional weight loss [17,18]. For instance, 12-months after termination of liraglutide treatment adults with obesity gained on average, ∼2.5 kg of lean mass and ∼6.3 kg of fat mass (9.6 kg overall bodyweight gain) [18]. Similarly, postmenopausal women who gained on average, 3.65 kg in weight 12-months following cessation of weight loss experienced a mean 2.52 kg increase in fat mass with a 0.12 kg reduction in lean mass [17]. Consequently, GLP-1 RA cessation may precipitate rapid fat regain exceeding lean mass recovery gains, exacerbating sarcopenic obesity risk especially in adults with low initial muscle reserves (Fig. 1). Given the potential adverse consequences of sarcopenic obesity there is a need to educate healthcare professionals about the risks and to incorporate lifestyle interventions to prevent gains in fat and optimise muscle mass following GLP-1 RA cessation.

## 5 Conclusion

GLP-1 RAs offer a revolutionary weight loss strategy for obesity, yet the associated muscle loss raise concerns about their potential to induce sarcopenic obesity in older adults, especially following weight cycling with GLP-1 RAs. Specifically, significant muscle losses during treatment, coupled with disproportionate fat regain post-cessation, may accelerate sarcopenic obesity in individuals with limited muscle reserves, worsening functional and metabolic outcomes. Lifestyle interventions, particularly resistance exercise with adequate protein intake, have shown promise for preserving muscle during weight loss interventions and following discontinuation [19,20], but their effectiveness with GLP-1 RA therapies and in older adults either during or following cessation of treatment remains unclear. This highlights the need for further longitudinal research in those using GLP-1 RA therapies (including the impact of different types and doses of treatment) using state-of-the-art imaging technique (e.g., MRI or CT) to accurately capture changes in ‘lean muscle mass’ and elucidate body composition trajectories as well as more fundamental research to understand the mechanisms underlying such changes. This is important to optimize future preventative strategies, ensuring these therapies do not inadvertently shift older adults with obesity towards a sarcopenic obesity phenotype.

## Declaration of competing interest

The authors declare that they have no known competing financial interests or personal relationships that could have appeared to influence the work reported in this paper.
